# rnaCrosslinkOO: an object-oriented R package for the analysis of RNA structural data generated by RNA crosslinking experiments

**DOI:** 10.1093/bioinformatics/btae193

**Published:** 2024-04-10

**Authors:** Jonathan L Price, Omer Ziv, Malte L Pinckert, Andrew Lim, Eric A Miska

**Affiliations:** Department of Biochemistry, University of Cambridge, Cambridge, CB2 1GA, United Kingdom; Department of Biochemistry, University of Cambridge, Cambridge, CB2 1GA, United Kingdom; Eleven Therapeutics, Cambridge, CB2 0RE, United Kingdom; Department of Pathology, University of Cambridge, Cambridge, CB2 1QP, United Kingdom; Department of Biochemistry, University of Cambridge, Cambridge, CB2 1GA, United Kingdom; Department of Biochemistry, University of Cambridge, Cambridge, CB2 1GA, United Kingdom

## Abstract

**Summary:**

RNA (ribonucleic acid) molecules have secondary and tertiary structures in vivo which play a crucial role in cellular processes such as the regulation of gene expression, RNA processing and localization. The ability to investigate these structures will enhance our understanding of their function and contribute to the diagnosis and treatment of diseases caused by RNA dysregulation. However, there are no mature pipelines or packages for processing and analyzing complex *in vivo* RNA structural data. Here, we present rnaCrosslinkOO (RNA Crosslink Object-Oriented), a novel software package for the comprehensive analysis of data derived from the COMRADES (Crosslinking of Matched RNA and Deep Sequencing) method. rnaCrosslinkOO offers a comprehensive pipeline from raw sequencing reads to the identification and comparison of RNA structural features. It includes read processing and alignment, clustering of duplexes, data exploration, folding and comparisons of RNA structures. rnaCrosslinkOO also enables comparisons between conditions, the identification of inter-RNA interactions, and the incorporation of reactivity data to improve structure prediction.

**Availability and implementation:**

rnaCrosslinkOO is freely available to noncommercial users and implemented in R, with the source code and documentation accessible at https://CRAN.R-project.org/package=rnaCrosslinkOO. The software is supported on Linux, macOS, and Windows platforms.

## 1 Introduction

RNA molecules exhibit secondary and tertiary structures *in vivo*. While ribosomal RNA (rRNA) with secondary structure and base pairings between nucleotides is a familiar concept, mRNA is frequently represented visually as a linear entity, typically marked with 5′ and 3′ labels (Vicens and Kieft 2022). This bias in conceptualization, compounded by the complexities of investigating RNA structures *in vivo* has led to the study of RNA structure lagging behind other fields of structural biology.

RNA structure is observed as dynamic *in vivo*, adapting to localized spatiotemporal conditions within the cell (Solayman *et al.* 2022). Factors such as minor changes in pH, salt concentrations, ligand availability, temperature, or point mutations can influence the behavior of covalent base pairs, consequently affecting the structure (Wan *et al.* 2011). These structural changes have a diverse impact on cellular biology (Mortimer *et al.* 2014), including transcriptional regulation (Tsai *et al.* 2010), splicing (Kar *et al.* 2011), translation (Ray *et al.* 2009), and RNA decay (Fukuchi and Tsuda 2010).

Studying RNA structure *in vivo* is becoming a combinatorial assay with recent success in the field coming from utilizing psoralen crosslinking methods, chemical probing and *in silico* folding for the same RNA (Spitale and Incarnato 2023). This is because the limitation of each of the methods are mitigated by the others; psoralen crosslinking methods such as COMRADES (Crosslinking of Matched RNA and Deep Sequencing) (Ziv *et al.* 2018, 2020), PARIS (Lu *et al.* 2018), SPLASH (Aw *et al.* 2016), Karr-Seq (Wu *et al.* 2024), and LIGR-seq (Sharma *et al.* 2016) provide evidence for long-range base-pairing although not at base-pair resolution which complicates the use of *in silico* folding methods. Chemical probing methods, such as icSHAPE (Flynn *et al.* 2016), when applied alone, are limited by the presence of RNA binding proteins, solvent accessibility and their inability to detect long-range base pairing. However, their ability to provide information at single nucleotide resolution can improve *in silico* folding predictions of RNA crosslinking data. The subsequent analysis of this combinatorial data is complicated.

We present the rnaCrosslinkOO (RNA Crosslinking Object-Oriented) R package, a novel and versatile R package, that focusses on the downstream analysis of RNA crosslinking data. Analysis strategies exist for analyzing RNA crosslinking data including CRSSNT (Gabryelska *et al.* 2022, Zhang *et al.* 2022). Their focus is on the alignment of the chimeric reads and integrate well with this R package that provides visualization and analysis of processed reads. Although the package was designed to analyze COMRADES data, the rnaCrosslinkOO R package will accept any crosslinking data presented in the correct format. The object-oriented nature of the package allows the storage of raw and processed data.

## 2 Methods and application

### 2.1 Read pre-processing

The COMRADES experimental protocol results in high-throughput sequencing data in FASTQ format. To process these raw sequencing reads for downstream analysis with the rnaCrosslinkOO package, we have developed a Nextflow (Di Tommaso *et al.* 2017) pipeline. Parameters for steps in the Nextflow pipeline can be found in Supplementary Table S3 (https://github.com/JLP-BioInf/rnaCrosslinkNF). Crosslinking experiments have varied library preparation protocols and often small differences mean that it is not possible to follow a prescribed pipeline for data pre-processing. For this reason, users can also create their own input files provided they follow the guidelines set out in the vignette and Supplementary Table S4.

### 2.2 The rnaCrosslinkOODataSet object

The rnaCrosslinkOO package can be installed using *install.packages* in R. A full vignette and usage documentation can be found on the CRAN website (https://CRAN.R-project.org/package=rnaCrosslinkOO) and through the *vignette* function. The object-oriented R package centers around a new S4 class, the *rnaCrosslinkDataSet*. This class consists of slots that facilitate the storage and accessibility of the data.

### 2.3 Reading in and exploring the global interactions

**Figure 1.**
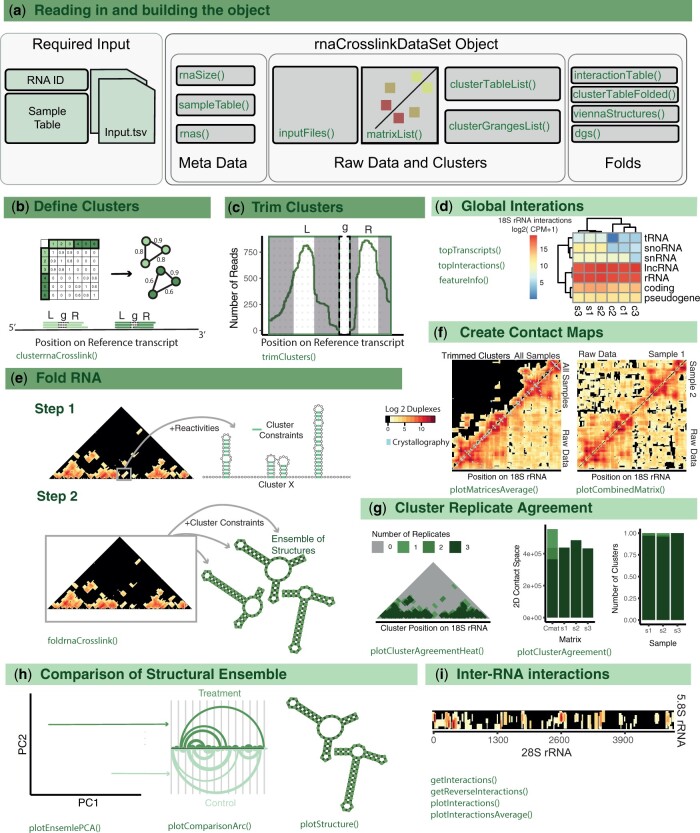
*rnaCrosslinkOO*. Main steps of the algorithm are shown in a,b, and c and optional analysis steps are shown in d-i. (a) Input for rnaCrosslinkOO and the main slots of the rnaCrosslinkDataSet object displayed with grey boxes with the functions used to access the slots in green. *sampleTable—*A table with the following column names; file (file path for sample), group (a code for the sample group), sample (a numeric value showing which replicates belong to which group), sampleName (a unique sample identifier). *inputFiles, matrixList*—The *inputFiles* slot contains the duplexes in their original input format (Supplementary Table S3) and the *matrixList* contains the data as contact matrices. *clusterGRangesList, ClusterTableList—*These two slots are related to the clustering of the duplexes and contain a GRanges object and data frame of the cluster coordinates. *viennaStructures, dgs, clusterTableFolded, and interactionTable—*These slots relate to the folding of the RNA. The *viennaStructures* and *dgs* slots contain the vienna format structures and the free energy value for the predicted structures for each sample. The *interactionTable* contains the constraints identified from folding each of the clusters and the *clusterTableFolded* slot contains the predicted fold for each cluster. (a–i) Schematics and examples of steps in the rnaCrosslinkOO package, further detail for each step can be found in the main text.

Loading the data into the rnaCrosslinkOO package requires; (i) Sample metadata in the form of a tab-separated table. (ii) The output of the COMRADES Nextflow pipeline or files in the same format (Supplementary Table S3). (iii) The ID of the RNA of interest. The COMRADES experimental protocol involves a round of enrichment for a specific RNA. However, the resulting data also contains structural information from other RNAs, as well as inter- and intra-RNA interactions for the RNA of interest. To gain a comprehensive overview of the RNAs within the dataset, there are three primary methods: *featureInfo*, *topTranscripts*, and *topInteractions*. These methods present the user with a table showing highly abundant RNAs and RNA-RNA interactions in the dataset (Fig. 1d).

### 2.4 Exploring the inter-RNA interactions

The COMRADES protocol crosslinks any nucleotide bound RNAs, this includes inter-RNA interactions. Exploring these interactions after using the *topInteractions* and *topInteractors* methods can be performed with *getInteractions* and *getReverseInteractions*. Plotting the resultant tables shows the location of reads for another chosen transcript. Users can also explore inter-RNA interactions as a 2D contact map with *plotInteractions* (Fig. 1i).

### 2.5 Exploring the intra-RNA interactions

#### 2.5.1 Clustering and trimming duplexes

In the COMRADES data, crosslinking and fragmentation leads to the production of redundant structural information, where the same *in vivo* structure from different RNA molecules produces slightly different RNA fragments. Clustering of these duplexes that originate from the same place in the reference transcript reduces computational time during the folding step and allows trimming of these clusters to improve the resolution. Clustering is performed as described in Ziv *et al.* (2020). Briefly, gapped alignments can be described by the transcript coordinates of the left (L) and right (R) side of the reads and by the nucleotides between L and R (*g*). Reads with similar or identical *g* values are likely to originate from the same structure of different molecules. In rnaCrosslinkOO, an adjacency matrix is created for all chimeric reads based on the nucleotide difference between their g values. From these weights the network can be defined as: G = (V, E). To identify clusters within the graph, the graph is clustered using random walks with the *cluster_waltrap* function (steps = 2) from the iGraph package (Csárdi *et al.* 2023). These clusters often contain a small number of longer L or R sequences due to the random fragmentation in the COMRADES protocol. Given the assumption that the reads within each cluster likely originate from the same structure in different molecules these clusters can be trimmed to contain the regions from L and R that have the most evidence (Fig. 1b and c). The clustering and trimming is achieved with the *clusterrnaCrosslink* and *trimClusters* methods. The cluster agreement between replicates can be inspected with *plotClusterAgreement* and *clusterAgreementHeat* (Fig. 1g).

#### 2.5.2 Check for domains

Folding RNA *in silico* becomes more computationally expensive and inaccurate as the size of the RNA increases. To allow the user to fold smaller parts of the RNA of interest rnaCrosslinkOO employs the *plotDomains* method. In the analysis of Hi-C data, domains are used to compartmentalize areas of the DNA with high inter-domain interactions and less interactions outside of the domain. Here we utilize a package designed for Hi-C analysis, TopDom to achieve this effect (Shin *et al.* 2016). The package was designed for larger molecules and the function provides output for a range of parameters (Supplementary Fig. S1B).

#### 2.5.3 Folding

After choosing a domain, the user can create predicted structures for any region or the whole RNA of interest using the *foldrnaCrosslink* method. The folding works as follows; firstly, all clusters in the region are folded *in silico* using RNAFold from the Vienna package (Lorenz *et al.* 2011). For short range clusters (*g *>* *10 nt) this is done by folding the region with RNAFold. For long-range clusters, an artificial linker is created between the two sides of the cluster and this sequence is folded using RNAFold. From these predicted structures of the clusters, the nucleotide contacts are then stored as constraints for the next step in the folding (Fig. 1e). Due to alternative topologies of the RNA *in vivo*, some of the cluster constraints may be mutually exclusive. In step two, the transcript region is folded 100 times by default, to produce a representative structural ensemble. Each time the RNA is folded, hard constraints that were identified in the first step are added sequentially and each time a constraint is added, the RNA is refolded. In the case where a constraint that is added shares a nucleotide position with a previously added constraint this new constraint is simply removed, and a new constraint is added. The user specifies how many constraints are added to each of the folded molecules. This produces an ensemble of structures that is stored in the object (Fig. 1e). To aid the analysis of the representative structural ensemble there are three functions; *plotEnsemblPCA, plotComparisonArc, structurePlot* (Fig. 1h). Although it is not yet common place to analyze chemical probing data with RNA crosslinking data, and it is still unclear how to integrate this disparate datasets, there is an option to include chemical probing data in this step.

### 2.6 Usage of rnaCrosslinkOO on an un-enriched dataset

To demonstrate the functionality of rnaCrosslinkOO, Fig. 1Supplementary Figs S1 and S2 show the analysis of EEF1A1P5 and the 18S ribosomal rRNA (18S rRNA). Firstly, EEF1A15P, Supplementary Figure S1A shows the contact maps for the three replicates, trimmed clusters and raw data. Supplementary Figure S1C shows the combinations of the trimmed clusters for all replicates and on the bottom half the agreement between the replicates. Domain identification of this RNA shows two domains, one large domain and a small domain on the 3ʹ end (Supplementary Fig. S1B). We folded the whole RNA ten times using constraints with at least ten supporting reads. These constraints originated from interactions ST1, ST2, ST3, and ST4 which are highlighted in Supplementary Fig. S1A. We also folded the whole RNA using RNAFold with no constraints (Supplementary Fig. S1D). We find that the COMRADES experimental evidence supports the RNAFold prediction of ST1 and ST3 which ensure a circularized RNA, while ST2 and ST4 do not appear in the MFE structure using RNAFold alone and could represent functional RNA structures.

Secondly, for the 18S rRNA, Fig. 1f shows the trimmed clusters and raw data for the three samples, crystallography base pairs are shown with blue points. The contact maps for the three biological replicates separately can be found in Supplementary Fig. S2C. Figure 1g shows the agreements between the trimmed clusters of the 18S rRNA with many of the clusters showing agreement between the three replicates (>95%). Domain identification identifies several domains in the 18S RNA which can be taken through to the folding step, and these agree with the crystallography structure (Ban *et al.* 2000) (Supplementary Fig. S2B, Supplementary Table S2B). Clustering of the data identified 83, 79, and 85 trimmed clusters for samples 1, 2, and 3, respectively, with 78%, 77%, and 80% able to be explained by Watson and Crick base pairs in the crystallography structure (these are clusters existing in the same place as at least one interaction from the crystal structure) (Supplementary Fig. S2C and Supplementary Table S2A). The 18S was split into four segments before folding and for each sample the segment was folded five times (Supplementary Fig. S2D). The sensitivity and specificity (the number of true positives out of the total in the in crystal structure and the number of true positives out of the total in the predicted structure) was calculated for each folded RNA. The 18S domains fold with a range of sensitivity (Supplementary Fig. S2D). The 3ʹ domain has the highest accuracy when folding and contains >90% of the Watson-Crick base pairs identified in the crystallography structure. 5ʹ however has a structure with only 25% of interactions discovered. In each domain the predictions have a higher sensitivity when compared to using RNAFold alone (5ʹ—13%, C—19%, 3ʹM—18%, 3ʹm—79%) Supplementary Table S2C. The PCA in Supplementary Fig. S2E shows the different structures in the representative structural ensemble for the three samples. The structure highlighted with a grey box in Supplementary Fig. S2F is very similar to the canonical structure of the 3ʹm domain which has been predicted by rnaCrosslinkOO. The families of RNA the 18S RNA interacts with can be seen in Fig. 1d with the specific interactions of the 28S and 18S in Supplementary Fig. S2A and the 28S and 5.8S in Fig. 1i. These specific inter-RNA interactions do not agree with the crystal structures. A subsetted version of this dataset is supplied with the package and the commands used in this analysis are available within the vignette of the package. The full dataset is available on GEO (GSE246412) and other COMRADES datasets can be found in previous publications (Ziv *et al.* 2018, 2020).

## 3 Conclusion

The rnaCrosslinkOO R package complements current pipelines by providing infrastructure for the downstream analysis of RNA crosslinking experiments. Current analysis packages lack visualizations and ease of use. This package solves these problems by centring around a new class, the rnaCrosslinkDataSet. This allows for the different data types to be stored at each stage in the analysis. There are significant challenges in the analysis of RNA crosslinking data, such as: How can constraints derived from RNA crosslinking experiments be best combined with silico folding models? Also, to date, no RNA crosslinking and chemical probing data has been created from the same sample, so how can chemical probing data be best integrated into this *in silico* model? This at present is not clear. We hope providing a framework for the analysis of this data will allow for easier exploration of these questions and will ensure that RNA crosslinking experiments are more accessible and widely adopted.

## Supplementary Material

btae193_Supplementary_Data
