# Supplementary material for: rnaCrosslinkOO: an object-oriented R package for the analysis of RNA structural data generated by RNA crosslinking experiments
Source: Bioinformatics. 2024 Apr 10;40(4):btae193. doi: 10.1093/bioinformatics/btae193 (PMC11060868; doi:10.1093/bioinformatics/btae193)
Supplement: btae193_Supplementary_Data [file btae193_supplementary_data.docx]

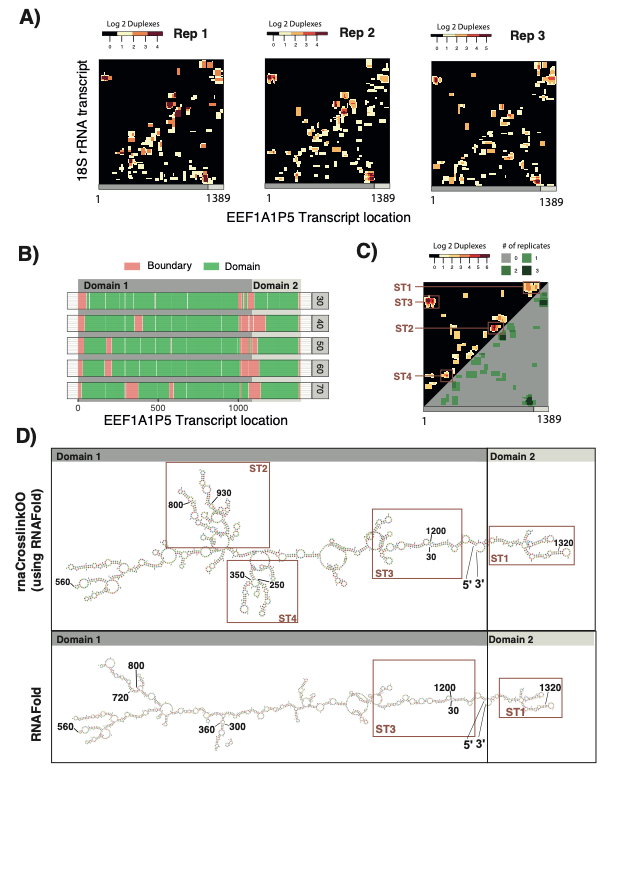


*Supplemental Figure 1.* **Example analysis of EEF1A1P5. A)** Contact maps for 3 replicates, showing raw data on the bottom triangle and trimmed clusters on the top triangle. **B)**  Domain identification for EEF1A1P5 showing two domains. **C)** Combined contact map showing the addition of the clusters for all 3 replicates on the top triangle and the cluster agreement between replicates on the bottom triangle. Clusters ST1, ST2, ST3, ST4 that pass the evidence cu-toff of 10 are highlighted in red. **D)** The predicted structure of EEF1A1P5 using rnaCrosslinkOO (on top) and with no constraints using RNAFold alone (on the bottom). The clusters highlighted in C) are again highlighted.

*Suppplementary Table 1.* **Cluster positions identified for EEF1A1P5.** The clusters are highlighted on Supplemental figure 1 C and D. They are the clusters with at least 10 supporting reads that went onto provide constraints in the folding of EEF1A1P5 (Supplemental Figure 1D)

| **Cluster ID** | **Left**  **Start** | **Left End** | **Right Start** | **Right End** | **Reads** | **sample** | **Supp Figure C,D ref** |
| --- | --- | --- | --- | --- | --- | --- | --- |
| 1 left | 21 | 115 | 1144 | 1229 | 26 | s1 | ST1 |
| 1 left | 21 | 78 | 1127 | 1216 | 44 | s3 | ST1 |
| 3 left | 27 | 75 | 1101 | 1209 | 14 | s2 | ST1 |
| 1 left | 28 | 134 | 1101 | 1211 | 38 | s2 | ST1 |
| 2 left | 33 | 80 | 1163 | 1215 | 12 | s2 | ST1 |
| 3:binLong:1 left left | 199 | 282 | 322 | 388 | 20 | s1 | ST4 |
| 4:binLong:1 left left | 228 | 289 | 315 | 365 | 10 | s2 | ST4 |
| 2:binLong:1 left left | 752 | 824 | 841 | 915 | 18 | s1 | ST2 |
| 20:binLong:1 left left | 756 | 833 | 846 | 915 | 10 | s2 | ST2 |
| 1:binLong:1 left left | 759 | 803 | 846 | 916 | 16 | s3 | ST2 |
| 3:binLong:1 left left | 760 | 804 | 829 | 915 | 14 | s2 | ST2 |
| 6:binLong:1 left left | 791 | 862 | 964 | 1028 | 16 | s1 | ST2 |
| 11:binLong:1 left left | 1146 | 1219 | 1290 | 1386 | 10 | s3 | ST3 |
| 9:binLong:1 left left | 1235 | 1288 | 1315 | 1377 | 10 | s2 | ST3 |
| 8:binLong:1 left left | 1173 | 1211 | 1227 | 1347 | 6 | s3 | ST3 |
| 22:binLong:1 left left | 1204 | 1270 | 1291 | 1371 | 8 | s1 | ST3 |


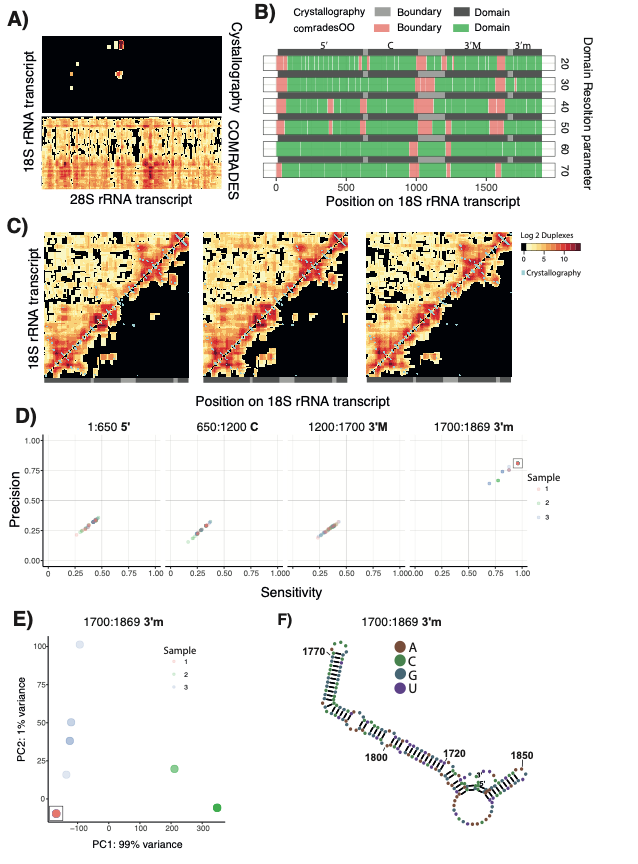


*Supplemental Figure 2.* ***Example of rnaCrosslinkOO analysis for the Human 18S rRNA, comparisons made with crystallography structure*** (Ban *et al.*, 2000). **A)** Inter-RNA interactions between the 28S rRNA and the 18S rRNA. **B)** Domain identification for the 18S rRNA. Grey background boxes show the domains identified in the crystallography structure **C)** Contact maps showing intra-RNA interactions for the 3 replicates for the 18S RNA, raw data and trimmed clusters. **D)** The precision and sensitivity of each samples predicted ensemble compared to the contacts in the crystallography structure. **E)** The output from *plotEnsemlePCA,* grey box highlights the structure drawn in F*.* ***F)*** The output from the *plotStructure* function*.*

*Supplementary table 2A*. **Cluster Numbers of 18S rRNA.**

| **group** | **sample** | **sampleName** | **rna** | **original** | **superClusters** | **trimmedClusters** | **novel** | **known** | **% known** |
| --- | --- | --- | --- | --- | --- | --- | --- | --- | --- |
| s | 1 | s1 | RN18S1_rRNA | 125 | 83 | 83 | 18 | 65 | 0.78313253 |
| c | 1 | c1 | RN18S1_rRNA | 68 | 62 | 62 | 16 | 46 | 0.74193548 |
| s | 2 | s2 | RN18S1_rRNA | 144 | 79 | 79 | 18 | 61 | 0.7721519 |
| c | 2 | c2 | RN18S1_rRNA | 63 | 59 | 59 | 13 | 46 | 0.77966102 |
| s | 3 | s3 | RN18S1_rRNA | 133 | 85 | 85 | 17 | 68 | 0.8 |
| c | 3 | c3 | RN18S1_rRNA | 74 | 66 | 66 | 15 | 51 | 0.77272727 |

*Supplementary Table 2B.* **Output of plotDomains for 18S RRNA.**

| **from.id** | **from.coord** | **to.id** | **to.coord** | **tag** | **size** | **sample** | **window** |
| --- | --- | --- | --- | --- | --- | --- | --- |
| 176 | 1750 | 341 | 3410 | domain | 1660 | c1 | 60 |
| 342 | 3410 | 461 | 4610 | domain | 1200 | c1 | 60 |
| 462 | 4610 | 599 | 5990 | domain | 1380 | c1 | 60 |
| 600 | 5990 | 791 | 7910 | domain | 1920 | c1 | 60 |
| 792 | 7910 | 949 | 9490 | domain | 1580 | c1 | 60 |
| 950 | 9490 | 1011 | 10110 | boundary | 620 | c1 | 60 |
| 1012 | 10110 | 1210 | 12100 | domain | 1990 | c1 | 60 |
| 1211 | 12100 | 1248 | 12480 | boundary | 380 | c1 | 60 |
| 1249 | 12480 | 1615 | 16150 | domain | 3670 | c1 | 60 |
| 1616 | 16150 | 1709 | 17090 | domain | 940 | c1 | 60 |
| 1710 | 17090 | 1849 | 18490 | domain | 1400 | c1 | 60 |
| 1850 | 18490 | 1898 | 18980 | gap | 490 | c1 | 60 |
| 1 | 0 | 60 | 600 | boundary | 600 | c1 | 50 |
| 61 | 600 | 177 | 1770 | domain | 1170 | c1 | 50 |
| 178 | 1770 | 224 | 2240 | domain | 470 | c1 | 50 |
| 225 | 2240 | 376 | 3760 | domain | 1520 | c1 | 50 |
| 377 | 3760 | 401 | 4010 | boundary | 250 | c1 | 50 |
| 402 | 4010 | 456 | 4560 | domain | 550 | c1 | 50 |
| 457 | 4560 | 598 | 5980 | domain | 1420 | c1 | 50 |
| 599 | 5980 | 640 | 6400 | boundary | 420 | c1 | 50 |
| 641 | 6400 | 791 | 7910 | domain | 1510 | c1 | 50 |
| 792 | 7910 | 1011 | 10110 | domain | 2200 | c1 | 50 |
| 1012 | 10110 | 1113 | 11130 | boundary | 1020 | c1 | 50 |
| 1114 | 11130 | 1209 | 12090 | domain | 960 | c1 | 50 |
| 1210 | 12090 | 1250 | 12500 | boundary | 410 | c1 | 50 |
| 1251 | 12500 | 1354 | 13540 | domain | 1040 | c1 | 50 |
| 1355 | 13540 | 1523 | 15230 | domain | 1690 | c1 | 50 |
| 1524 | 15230 | 1583 | 15830 | boundary | 600 | c1 | 50 |
| 1584 | 15830 | 1625 | 16250 | boundary | 420 | c1 | 50 |
| 1626 | 16250 | 1713 | 17130 | domain | 880 | c1 | 50 |
| 1714 | 17130 | 1849 | 18490 | domain | 1360 | c1 | 50 |
| 1850 | 18490 | 1898 | 18980 | gap | 490 | c1 | 50 |

*Supplementary Table 2C.* **Precision and sensitivity for folding of 18S rRNA domains using RNAFold.**

| **Shared** | **Total prediction** | **total in crytal** | **FP** | **FN** | **TP** | **Specificity** | **Sensitivity** | **Coordinate** |
| --- | --- | --- | --- | --- | --- | --- | --- | --- |
| 102 | 609 | 454 | 507 | 352 | 102 | 0.22 | 0.1675 | 1:1896 |
| 27 | 205 | 142 | 178 | 115 | 27 | 0.19 | 0.1317 | 1:650 |
| 34 | 178 | 122 | 144 | 88 | 34 | 0.28 | 0.191 | 650:1200 |
| 29 | 154 | 125 | 125 | 96 | 29 | 0.23 | 0.1883 | 1200:1700 |
| 47 | 59 | 49 | 12 | 2 | 47 | 0.96 | 0.7966 | 1700:1869 |

*Supplementary Table 3.* **Parameters and programs for read pre-processing.**

| **Program** | **Step** | **Parameters** |
| --- | --- | --- |
| cuatadapt 1 | 1 | -b AGATCGGAAGAGCACACGTCTGAACTCCAGTC, -B AGATCGGAAGAGCGTCGTGTAGGGAAAGAGTGT |
| Pear | 2 | min-assembly-length 20, -v 7, and -j 5 |
| tstk collapse | 3 | --minreads 1 |
| cuatadapt 2 | 4 | -m 10, -u 6 |
| star | 5 | runThreadN 5, --genomeLoad NoSharedMemory, --outReadsUnmapped Fastx, --outFilterMultimapNmax 10000, --outFilterScoreMinOverLread 0, --outFilterMatchNminOverLread 0, --outSAMattributes All, --outSAMtype BAM SortedByCoordinate, --alignIntronMin 5, --alignIntronMax 3900000000, --seedSearchStartLmax 14, --seedSplitMin 14, --scoreGap 0, --scoreGapNoncan 0, --scoreGapGCAG 0, --scoreGapATAC 0, --scoreGenomicLengthLog2scale -0.1, --chimFilter None, --chimOutType WithinBAM HardClip, --chimSegmentMin 7, --chimJunctionOverhangMin 8, --chimScoreJunctionNonGTAG 0, --chimScoreDropMax 150, and --chimMainSegmentMultNmax 10000 |

*Supplementary Table 4.* **Column specifications for input files.**

| **Column Number** | **Description** | **Notes** |
| --- | --- | --- |
| 1 | Read Name | - |
| 2 | Read Sequence | - |
| 3 | Side 1 transcript ID | Works best if “_” delimited and the last segment is the RNA species |
| 4  5 | NA – empty space  Side 1 Position start in read | - |
| 6 | Side 1 Position end in read sequence | - |
| 7 | Side 1 Coordinate start in transcript | - |
| 8 | Side 1 Coordinate end in transcript | - |
| 9 | NA – empty space | This should be "." for all rows (room for something else) |
| 10 | Side 2 transcript ID | Works best if _ delimited and the last segment is the RNA species |
| 11 | Side 2 Position start in read sequence | - |
| 12 | Side 2 Position end in read sequence | - |
| 13 | Side 2 Coordinate start in transcript | - |
| 14 | Side 2 Coordinate end in transcript | - |
| 15 | NA – empty space | This should be "." for all rows (room for something else) |
